# Non Mycobacterial Virulence Genes in the Genome of the Emerging Pathogen *Mycobacterium abscessus*

**DOI:** 10.1371/journal.pone.0005660

**Published:** 2009-06-19

**Authors:** Fabienne Ripoll, Sophie Pasek, Chantal Schenowitz, Carole Dossat, Valérie Barbe, Martin Rottman, Edouard Macheras, Beate Heym, Jean-Louis Herrmann, Mamadou Daffé, Roland Brosch, Jean-Loup Risler, Jean-Louis Gaillard

**Affiliations:** 1 EA3647, UFR de médecine Paris Ile-de-France Ouest, Université de Versailles St-Quentin, Garches, France; 2 Unité de Recherche Systématique, Adaptation, Evolution (UMR 7138), Université Pierre-et-Marie-Curie, Paris, France; 3 Génoscope, Evry, France; 4 Laboratoire de Microbiologie, Hôpital Ambroise Paré (Assistance Publique-Hôpitaux de Paris), Boulogne, France; 5 Institut de Pharmacologie et Biologie Structurale (UMR 5089), Toulouse, France; 6 Intitut Pasteur, UP Pathogénomique Mycobactérienne Intégrée, Paris, France; 7 Laboratoire Statistique et Génome, UMR CNRS-INRA 8071-1152, Evry, France; University of Hyderabad, India

## Abstract

*Mycobacterium abscessus* is an emerging rapidly growing mycobacterium (RGM) causing a pseudotuberculous lung disease to which patients with cystic fibrosis (CF) are particularly susceptible. We report here its complete genome sequence. The genome of *M. abscessus* (CIP 104536T) consists of a 5,067,172-bp circular chromosome including 4920 predicted coding sequences (CDS), an 81-kb full-length prophage and 5 IS elements, and a 23-kb mercury resistance plasmid almost identical to pMM23 from *Mycobacterium marinum*. The chromosome encodes many virulence proteins and virulence protein families absent or present in only small numbers in the model RGM species *Mycobacterium smegmatis*. Many of these proteins are encoded by genes belonging to a “mycobacterial” gene pool (e.g. PE and PPE proteins, MCE and YrbE proteins, lipoprotein LpqH precursors). However, many others (e.g. phospholipase C, MgtC, MsrA, ABC Fe(3+) transporter) appear to have been horizontally acquired from distantly related environmental bacteria with a high G+C content, mostly actinobacteria (e.g. *Rhodococcus* sp., *Streptomyces* sp.) and pseudomonads. We also identified several metabolic regions acquired from actinobacteria and pseudomonads (relating to phenazine biosynthesis, homogentisate catabolism, phenylacetic acid degradation, DNA degradation) not present in the *M. smegmatis* genome. Many of the “non mycobacterial” factors detected in *M. abscessus* are also present in two of the pathogens most frequently isolated from CF patients, *Pseudomonas aeruginosa* and *Burkholderia cepacia*. This study elucidates the genetic basis of the unique pathogenicity of *M. abscessus* among RGM, and raises the question of similar mechanisms of pathogenicity shared by unrelated organisms in CF patients.

## Introduction

Mycobacteria form a group of over one hundred species, ranging from harmless saprophytic organisms to major human pathogens. The well known pathogenic species, such as *Mycobacterium tuberculosis*, *Mycobacterium leprae* and *Mycobacterium ulcerans*, belong to the subgroup of slowly growing mycobacteria (SGM). By contrast, rapidly growing mycobacteria (RGM) —almost 60 species of which have been identified—usually live in the soil or water and only rarely cause human infections [1]. *Mycobacterium abscessus* is one of the few RGM able to infect humans and is undoubtedly the most frequently isolated and the most difficult to combat [2].

*M. abscessus* was first described by Moore and Frerichs in 1953 [3]. These authors reported the isolation of a previously unknown mycobacterium from a human knee infection with subcutaneous abscess-like lesions (type strain *M. abscessus* ATCC 19977T), hence the name “*abscessus*”. With the recognition of *Mycobacterium chelonei* (now *M. chelonae*) in 1972, these two RGM organisms were classified as two subspecies of the same species. Over two decades, they were collectively designated “*M. chelonae*”, or even grouped with the RGM *Mycobacterium fortuitum* under the designation “*M. fortuitum* complex” [4]. It was only in 1992 that *M. abscessus* was separated from *M. chelonae* [5], and this separation soon resulted in the recognition that *M. abscessus* has a particular pathogenicity in humans [6]. Very recently, *M. abscessus* itself (now *M. abscessus sensu lato*) was shown to consist of three species: *M. abscessus sensu stricto*, *M. massiliense* and *M. bolletii* [7], [8]. These species are very closely related and cause a similar spectrum of human infections [9], [10]. Thus, hereafter, unless otherwise stated, they will be collectively referred to as “*M. abscessus*”.

Following its recognition as a distinct entity, and the development of molecular methods of identification for mycobacteria, *M. abscessus* has emerged as an important human pathogen over the last 10 years, causing many more cases of infection than *M. chelonae* and *M. fortuitum*—historically the most important pathogenic RGM [2], [11]. *M. abscessus* is responsible for more than 80% of all pulmonary infections due to RGM in the United States and is associated with a much higher fatality rate than any other RGM [6]. *M. abscessus* lung infection usually, but not exclusively, develops in subjects with underlying lung disorders (e.g. bronchiectasis, cystic fibrosis [CF]) [6]. The infection of CF patients is becoming a major issue: *M. abscessus* is being recovered with increasing frequency from CF patients, including young children. It causes a serious, life-threatening lung disease and is responsible for disseminated, often fatal infections following lung transplantation [12]–[18]. *M. abscessus* is also a leading cause of sporadic and epidemic cases of skin and soft-tissue RGM infections, following the use of contaminated syringes or needles, and after plastic or cardiac surgery [19], [20]. *M. abscessus* is not only pathogenic, it is also one of the most antibiotic-resistant RGM species [1]. It is resistant to most disinfectants and biocides and thrives in the most hostile environments—a feature associated with its propensity to cause outbreaks of healthcare-associated disease [1].

The pathogenicity of *M. abscessus* has been investigated in recent studies in various cell and mouse models. *M. abscessus* is an intracellular bacterium able to grow in macrophages and free-living amebas [8], [21]. *M. abscessus* infection in mice is associated with granulomatous lesions spontaneously evolving toward caseous lesions [22]. Interferon gamma (IFN-γ) and tumor necrosis factor (TNF) are the key cytokines of the murine host response, and are absolutely required to control infection [22]. Studies have also identified major differences in pathogenic profile between the two forms in which *M. abscessus* is isolated from humans: the S (smooth) form and the R (rough) form [21]. The R form lacks a surface polyketide compound, glycopeptidolipid (GPL) [23], [24], and causes more severe infections in mice, strongly inducing TNF secretion by macrophages [23].

Over the last decade, genomic studies have shown how the ecological and pathogenic characteristics of certain SGM have changed through evolution. For example, *M. leprae*, the causal agent of leprosy, represents a model case of adaptation through massive genome reduction [25]. Gene deletion and decay have resulted in the elimination from *M. leprae* of many of the major metabolic activities present in the closely related species, *M. tuberculosis*, the tubercle bacillus. This process of gene deletion is associated with the divergent evolution of *M. leprae* towards an obligate intracellular lifestyle. Other mycobacteria have acquired plasmid-borne virulence factors. The presence of a giant plasmid involved in the synthesis of a potent macrolide toxin forms the basis, for example, of the unique pathogenic properties of *M. ulcerans*, the causal agent of Buruli ulcer [26]. Genomic studies have also revealed how the deletion of large chromosomal regions led to the attenuation of *Mycobacterium bovis* bacillus Calmette-Guérin, the only vaccine against tuberculosis currently available [27], [28].

Very few genomic studies have been performed in the RGM group, and none has dealt with a “pathogenic” RGM. The first RGM to be sequenced—*M. smegmatis*—is a model mycobacterium widely used in research laboratories as a surrogate host for the expression of heterologous mycobacterial genes. The other RGM organisms sequenced (e.g., *M. vanbaalenii*) have been studied because they are able to degrade polycyclic aromatic hydrocarbons and are therefore of potential interest for use in environmental bioremediation [29]. We report here the complete genome sequence of *M. abscessus* (*sensu stricto*) and the insights it has provided into the genetic basis of its the pathogenicity of this bacterium, which is highly unusual among RGM. Whole-genome analysis not only revealed the presence of many “mycobacterial” virulence genes, but also showed that *M. abscessus* had a large series of specific genes in common with two pathogens most frequently isolated from CF patients—*Pseudomonas aeruginosa* and *Burkholderia cepacia*. These genes were presumably acquired from distantly related environmental bacteria via horizontal gene transfer (HGT).

## Results

### The *M. abscessus* genome

#### General features and comparison with other Mycobacterium species

**Figure 1:**
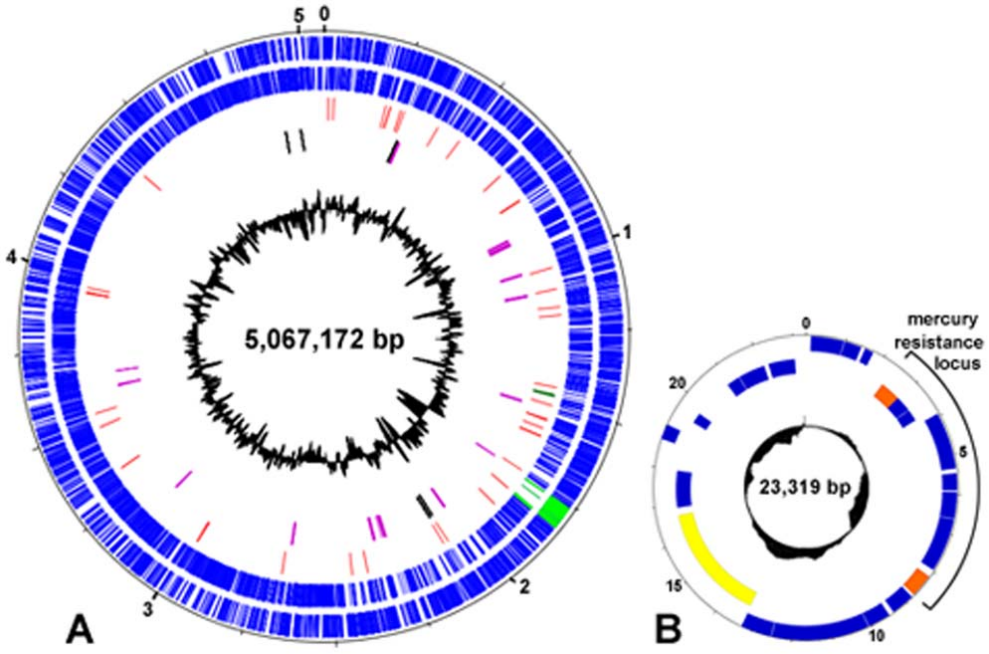
The *M. abscessus* CIP 104536T genome. (A) Circular representation of the chromosome. The initiation codon for the *dnaA* gene was chosen as the starting point for numbering. The scale is in Mb. Moving inward, the first two circles show forward and reverse genes (blue lines); light-green lines indicate phage genes. The third circle shows tRNA genes (red) and rRNA operon (dark-green). The fourth circle shows genes presumably acquired “en bloc” from non mycobacterial organisms by HGT (purple) and IS elements (black). The inner black histogram represents the local G+C content (scale: 50% to 69%). (B) Circular representation of the 23-kb mercury resistance plasmid. The scale is in kb. Forward and reverse genes and the local G+C content are indicated with the same code as for the chromosome map. The plasmid carries a mercury resistance operon flanked by two genes encoding site-specific recombinases (MAB_p04c and MAB_p10, orange); it also encodes a relaxase/helicase that may function in conjugation or mobilization (MAB_p15c, yellow).

The *M. abscessus* (*sensu stricto*) genome consists of a circular chromosome of 5,067,172 base pairs (bp) including 4,920 predicted coding sequences (CDS) with a coding capacity of 93%, and a G+C content of 64% other than in the prophage region (59.5%) (Table 1 and Fig. 1A). A circular 23 kb mercury resistance plasmid (23,319 bp; G+C content, 68%) was also detected (Fig. 1B) and shown to have a nucleotide sequence 99.9% identical to that of the 23 kb pMM23 mercury resistance plasmid from *Mycobacterium marinum* (strain ATCC BAA-535) [30]. Like pMM23, the 23 kb *M. abscessus* plasmid carries a mercury resistance operon flanked by two genes encoding site-specific recombinases; it also encodes a relaxase/helicase that may function in conjugation or mobilization (Fig. 1B).

**Table 1:** General features of the *M. abscessus* genome and comparison with other *Mycobacterium* species.

Features	RGM				SGM			
	Mabs	Msmeg(a)	*M. gilvum* (a)	Mvanba(b)	Mtb(c)	*M. avium* (a)	*M. marinum* (d)	*M. ulcerans* (e)
Genome size, bp	5,067,172	6,988,209	5,619,607	6,491,865	4,411,532	5,475,491	6,636,827	5,631,606
G+C content, %	64,1	67	67	67	65,6	68	65	65
Protein coding, %	93	90	92	91	90,8	88	89	72
Proteins	4920	6716	5241	5979	3959	5120	5424	4160
tRNAs	47	47	47	50	45	45	46	45
rRNA operons	1	2	2	2	1	1	1	1
Prophage elements, no.	4(f)	NR	NR	NR	2	NR	10	2
IS, total no. of copies	5	112	NR	NR	54	NR	7	302

(a) http://www.ncbi.nlm.nih.gov.

(b) [29].

(c) [31].

(d) [30].

(e) [87].

(f) Including the 81-kb full-length prophage and three prophage-like elements detailed in Table S1.

Abbreviations: RGM, rapidly growing mycobacteria ; SGM, slowly growing mycobacteria ; Mabs, *M. abscessus* ; Msmeg, *M. smegmatis*; Mvanba, *M. vanbaalenii*; Mtb, *M. tuberculosis*; nt, nucleotide; IS, insertion sequence; NR, not reported.

**Figure 2:**
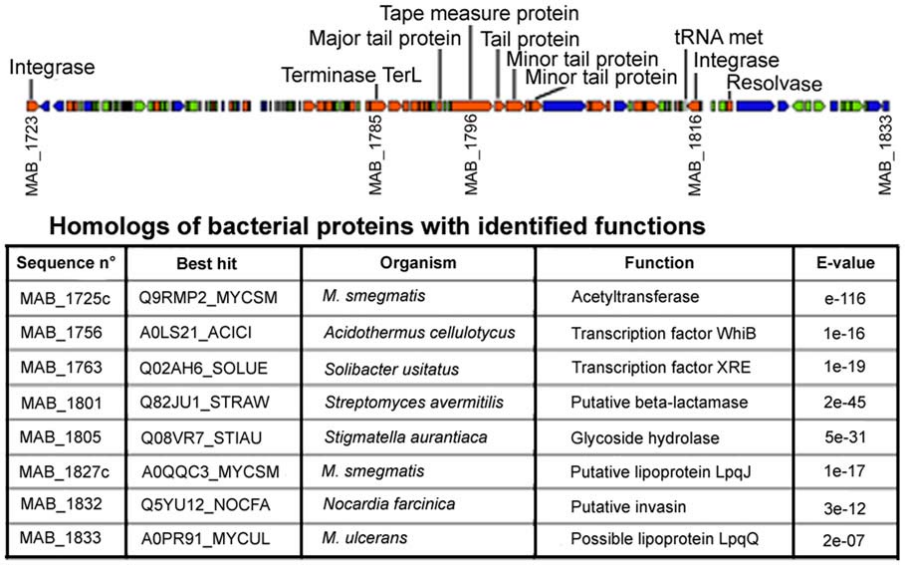
The *M. abscessus* full-length prophage. Each arrow represents a predicted protein-coding gene (length approximately to scale). Orange, similar to other phage proteins; blue, similar to other bacterial proteins; green, hypothetical protein. The table shows homologs of bacterial proteins with identified functions (Uniprot Blast search).

The *M. abscessus* chromosome is about 1.92 Mb smaller than the *M. smegmatis* genome; these two genomes are collinear, with no evidence of extensive rearrangements (Fig. S1). The *M. abscessus* chromosome includes 47 tRNA genes, but has a single ribosomal RNA operon, a feature of SGM genomes [31]. It contains a full-length prophage (81 kb) resembling the members of a recently characterized group of dsDNA tailed mycobacteriophages [32]. This prophage is integrated into a Met tRNA and contains 112 CDS, 8 (7.1%) of which are similar to bacterial proteins with identified functions (Fig. 2). There are also three prophage-like elements (Table S1). Unlike other sequenced mycobacteria, *M. abscessus* has very few insertion sequences (IS) in its genome: there are only five IS, each present as a single copy (Table 1). These elements include the composite element ISMab1 [33], which is probably part of an integrated plasmid (gene encoding a putative plasmid replication initiator protein in its vicinity [MAB_2100]).

#### Functional information

It was possible to assign a biological function to 60.5% of the CDS on the *M. abscessus* chromosome; 27.5% were found to be conserved hypothetical proteins and 12% were unique. The distributions of *M. abscessus* and *M. smegmatis* proteins, according to the Kegg classification, were different, with a lower proportion of *M. abscessus* proteins involved in xenobiotic biodegradation and metabolism (p<10^−4^, chi-squared test), and in biosynthesis of secondary metabolites (p = 0.02) (Table S2). Consistent with the smaller size of the *M. abscessus* genome, most paralogous families were found to be underrepresented in *M. abscessus* with respect to *M. smegmatis* (Table S3). This was particularly true for paralogs involved in the adaptation of microorganisms to diverse environments (e.g. ABC transporters, two-component sensor histidine kinases). Most of the small number of protein families found to be overrepresented in *M. abscessus* are known to be associated with mycobacterial pathogenicity (e.g. PE and PPE proteins, MCE and YrbE proteins, lipoprotein LpqH precursors, lipases/esterases/monooxygenases). Others, such as the members of the ArsC family, salicylate hydroxylases and cysteine desulfurases, are hallmarks of organisms living in soil or water.

#### Transfers of blocks of genes from non mycobacterial environmental organisms

**Figure 3:**
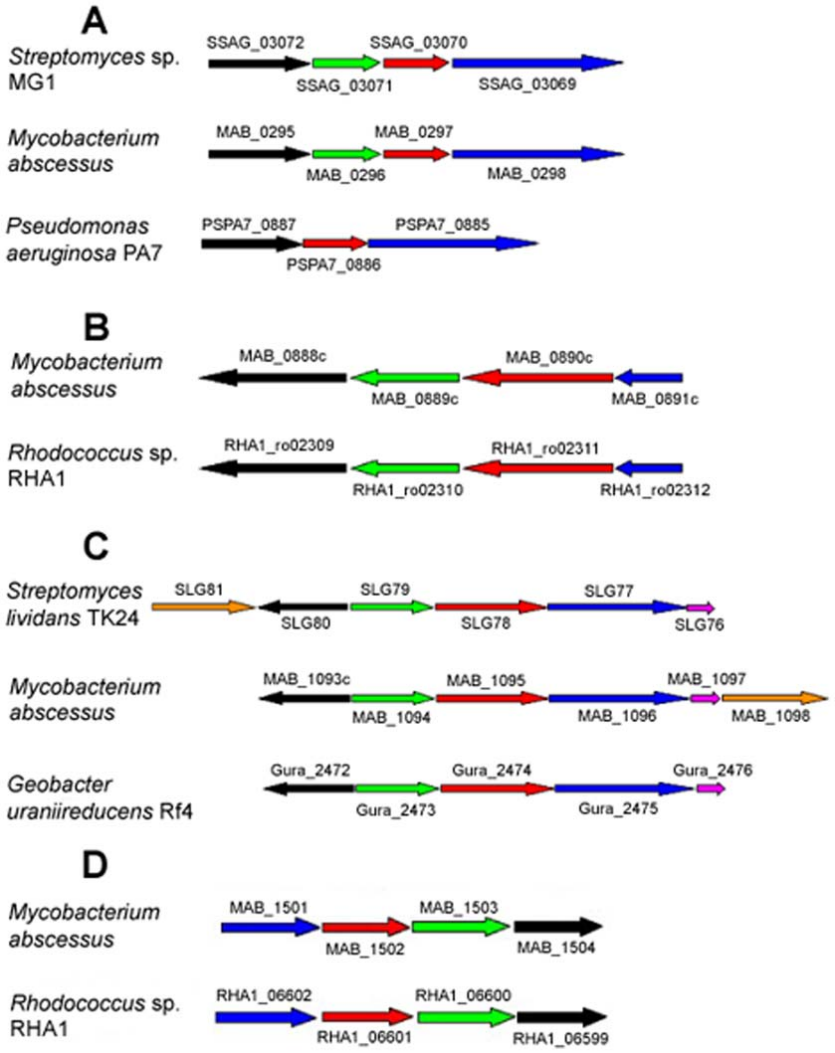
Examples of gene blocks presumably inherited from non mycobacterial organisms. (A) MAB_0295-0298 (phenazine biosynthesis). (B) MAB_0888c-0891c (homogentisate catabolism). (C) MAB_1093c-1098 (DNA degradation [*dnd* locus]). (D) MAB_1501-1504 (iron uptake). Genes are drawn approximately to scale and are indicated according to their names in the Embl-Ebi database.

We detected 17 gene clusters syntenic with regions from non mycobacterial organisms, and which are absent from other sequenced mycobacterial species (Tables 2 and S4), suggesting multiple *en bloc* HGTs. The factors encoded by these gene clusters are much more similar to proteins from syntenic organisms than to proteins from other mycobacteria, or have no significant mycobacterial homologs (Table S4). The hypothesis of multiple HGT of blocks of genes is also supported by significant differences in codon usage for proline and arginine between this subset of genes and other *M. abscessus* genes (p<10^−4^, chi-squared test). The organisms with the best conserved syntenies are all environmental bacteria with a high G+C content, mostly actinobacteria (*Rhodococcus* sp., *Streptomyces* sp., *Nocardia* sp.), but also *Pseudomonas* sp. and *Burkholderia* sp. (Tables 2 and S4). There are more horizontally acquired gene clusters in the 5′ half of the genome than in the 3′ half (Fig. 1), with most concentrated into two “hot-spots” (MAB_0888c-1098, MAB_2027-2286). Examples of such gene clusters are shown in Fig. 3.

**Table 2:** Gene clusters syntenic with non mycobacterial organisms and not observed in other mycobacteria(a).

Cluster no.	Mabs CDS.	Main synteny(b)	Putative function
1	MAB_0295-0298(c)	*Streptomyces* sp. MG1 (B4V5P4_9ACTO)(d)	Phenazine biosynthesis
2	MAB_0300c-0303	*Nocardia farcinica* (Q5YP17_NOCFA)	Resistance to salicylic acid-mediated defense mechanisms
3	MAB_0888c-0891c	*Rhodococcus* sp. (Q0SEC1_RHOSR)(e)	Homogentisate catabolic pathway
4	MAB_0899c-0911(f)	*Nocardia farcinica* (Q5YXU1_NOCFA)(g,h)	Phenylacetic acid degradation
5	MAB_1014c-1019c	*Rhodococcus* sp. (Q0S4M6_RHOSR)	Unknown
6	MAB_1093c-1098	*Streptomyces lividans* (Q460H9_STRLI)(i)	DNA degradation (*dnd* locus)
7	MAB_1501-1504	*Rhodococcus* sp. (strain RHA1) (Q0S261_RHOSR)(j)	Iron uptake
8	MAB_1720-1722	*Rhodococcus* sp. (Q0S6K3_RHOSR)	Two-component system
9	MAB_2027-2032	*Pseudomonas putida* F1 (A5W4P6_PSEP1)	Biosynthesis of phytotoxic peptides and antibiotics
10	MAB_2251-2253	*Burkholderia cepacia* complex (Q0B890_BURCM)	Unknown
11	MAB_2255-2257(k).	*Myxococcus xanthus* (Q1D6A2_MYXXD)	Polyketide biosynthesis
12	MAB_2257-2258(l,m)	*Streptomyces ambofaciens* (A3KI34_STRAM)	Polyketide biosynthesis
13	MAB_2278-2286(n).	*Streptomyces coelicolor* (Q9K3F5_STRCO)	Unknown
14	MAB_2610-2613	*Bacillus pumilus* (B4AMJ6_BACPU)	Carbohydrate transport
15	MAB_3112-3115	*Nocardia farcinica* (Q5YN04_NOCFA)	Unknown
16	MAB_3569c-3574c(o ,p)	*Streptomyces antibioticus* (Q0R4L5_STRAT)	Biosynthesis of secondary metabolites
17	MAB_3621c-3623	*Rhodococcus* sp. (Q0SA51_RHOSR)(q)	Taurine metabolism

(a) Clusters comprising ≥3 syntenic genes.

(b) The organism and the homolog of the 5′ *M. abscessus* gene product (in brackets, entry name in Swiss-Prot/TrEMBL database) are indicated.

(c) Upstream, MAB_0292c is homologous to an ISX08 transposase from *Saccharopolyspora erythraea*.

(d) Also A6UZN8_PSEA7-A6UZN6_PSEA7 from *Pseudomonas aeruginosa* (strain PA7) (see also Fig. 3).

(e) Partial synteny (MAB_0888c-0890c) in *B. cepacia*.

(f) Downstream, MAB_0920 is homologous to a phenylacetic acid-responsive trancriptional repressor gene from *Kineococcus radiotolerans*.

(g) Also Q0SCR6_RHOSR–Q0SCS7_RHOSR from *Rhodococcus* sp. (strain RHA1).

(h) Partial synteny (MAB_0906-0910) in *B. cepacia*.

(i) Also A5G4D2_GEOUR- A5G4D6_GEOUR from *Geobacter uraniireducens* (strain Rf4) (see also Fig. 3)

(j) Also syntenic regions in *B. cepacia* and in the pathogens *Salmonella* Paratyphi A, *Salmonella* Typhimurium, *Salmonella* Typhi, and *Burkholderia mallei*.

(k) Just upstream, MAB_2254c is homologous to a PPE protein from *Mycobacterium vanbaalenii*.

(l) This cluster has only two genes but is part of a composite region encoding various polyketide synthases (see upstream, MAB_2255-MAB_2257).

(m) Just downstream, MAB_2259 is homologous to a putative O-methyltransferase gene from *Myxococcus xanthus*.

(n) MAB_2284 (homologous to Q9K3G5) is also homologous to the protein PrnC gene from *Burkholderia cepacia*.

(o) Except MAB_3570c, which is homologous to a 4′-phosphopantetheinyl transferase gene from the Actinomycetales (A3R4S1_9ACTO).

(p) MAB_3574c is also homologous to a 3-oxoacyl-[acyl-carrier-protein] synthase III from *Frankia alni*.

(q) Partial synteny (MAB_3621c-MAB_3622c) in *B. cepacia*.

Abbreviations: Mabs, *M. abscessus*; CDS, coding sequence.

### Virulence factors involved in intracellular parasitism

Many factors known to be involved in *M. tuberculosis* virulence have orthologs in the *M. abscessus* genome and best hits with proteins from other mycobacteria (Table S5). In addition to these “mycobacterial” factors, the *M. abscessus* genome encodes a number of “non mycobacterial” factors known to play a major role in microbial pathogenesis.

### “Mycobacterial” factors

#### The PE- PPE and ESAT-6 families

The PE and PPE proteins, with their characteristic proline-glutamate (PE), or proline-proline-glutamate (PPE) N-terminal motifs, are often found associated with ESX gene clusters, which encode ATP-dependent specific secretion systems and are named after the 6 kDa early secretory antigenic target ESAT-6 [31], [34], [35]. There are three PE and six PPE genes in *M. abscessus*, and three ESX loci, all similar to the essential and highly immunogenic ESX-3 gene cluster of *M. tuberculosis*.

#### MCE and yrbE proteins

MCE (mammalian cell entry) proteins allow mycobacteria to invade host cells [36]. There are seven *mce* operons in *M. abscessus* and only four in *M. smegmatis*, one of which is interrupted by an IS element in *M. smegmatis* (not shown). It has recently been suggested that the number of *mce* operons may be related to pathogenicity in actinomycetes: there are six *mce* operons in *Nocardia farcinica*, one of the agents causing nocardiosis, whereas *Streptomyces avermitilis* and *Streptomyces coelicolor*, both nonpathogenic soil bacteria, each have only one copy of the *mce* operon [37].

#### LpqH-like proteins

LpqH, also known as the 19 kDa protein, is an immunodominant antigen recognized by T cells and sera from patients with tuberculosis [38]. *M. abscessus* possesses four genes encoding LpqH-like proteins, scattered throughout the genome, suggesting that these molecules may be involved in the pathogenicity of *M. abscessus*, possibly through modification of the host response.

#### Regulators of virulence factors

*M. abscessus* has homologs of a large number of regulators known to control virulence factors in *M. tuberculosis*. Most appear to have counterparts in *M. smegmatis*, but there are a few exceptions, consistent with the specialization of *M. abscessus* towards pathogenicity. For example, *M. abscessus* possesses homologs of the five sigma factors shown to be involved in *M. tuberculosis* virulence (SigA, SigC, SigD, SigE, SigH), whereas *M. smegmatis* has homologs of only four of these factors (SigA, SigD, SigE, SigH). *M. abscessus* also has a protein homologous to the VirS virulence transcription factor of *M. tuberculosis*, whereas *M. smegmatis* does not.

### “Non mycobacterial” factors

#### Phospholipase C

**Figure 4:**
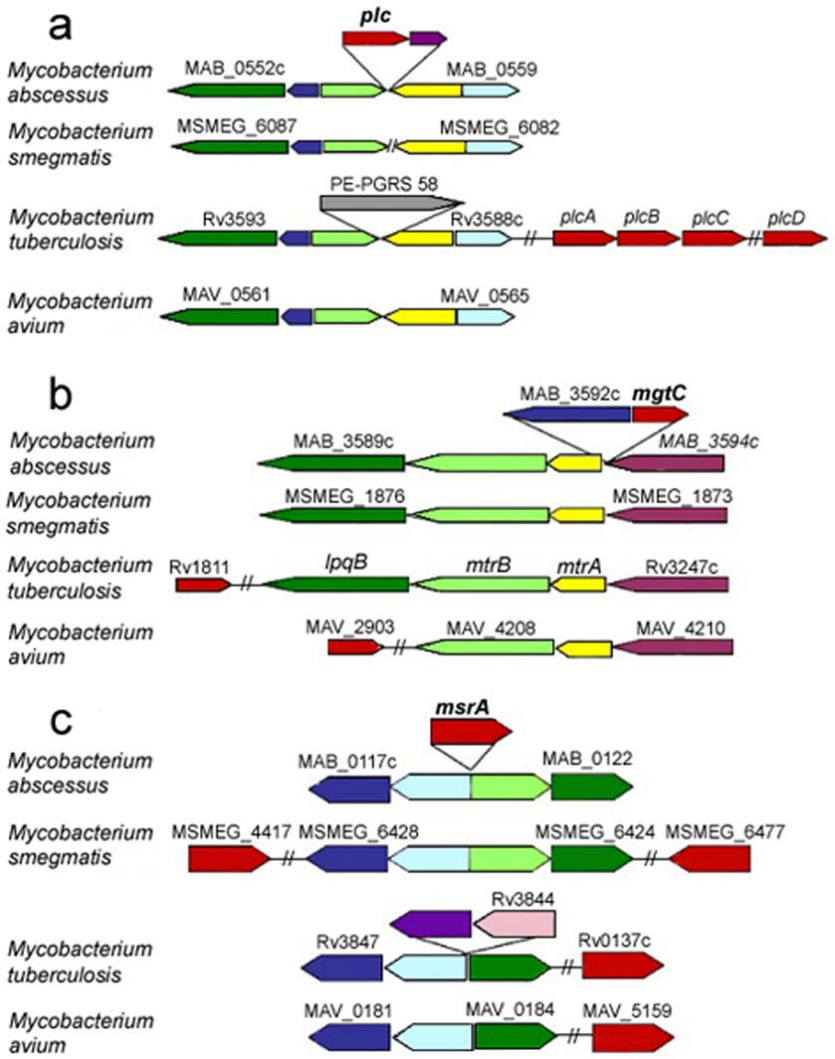
*plc*, *mgtC* and *msrA* loci in *M. abscessus*: comparison with other mycobacteria. a. *plc*: note that MAB_0557 (transcriptional regulatory protein AraC) has no homolog at the counterpart of the *M. abscessus plc* locus in the other mycobacterial species, suggesting an insertion; also note the substitution for a PE-PGRS gene in the corresponding *M. tuberculosis* region. b. *mgtC*: note that the gene encoding MgtC is located at other genomic sites in *M. tuberculosis* and *M. avium*; also note that MAB_3592c (probable chain fatty acid-CoA ligase, blue) has no homolog at the counterpart of the *M. abscessus mgtC* locus in the other mycobacterial species, also suggesting an insertion. c. *msrA*: note that MsrA-encoding genes are located at other genomic sites in other mycobacteria; and the presence of *sodA* (light blue) upstream of *M. abscessus msrA*; there is a substitution for a transposase gene (Rv3844) in the corresponding *M. tuberculosis* region. Out of scale.

**Figure 5:**
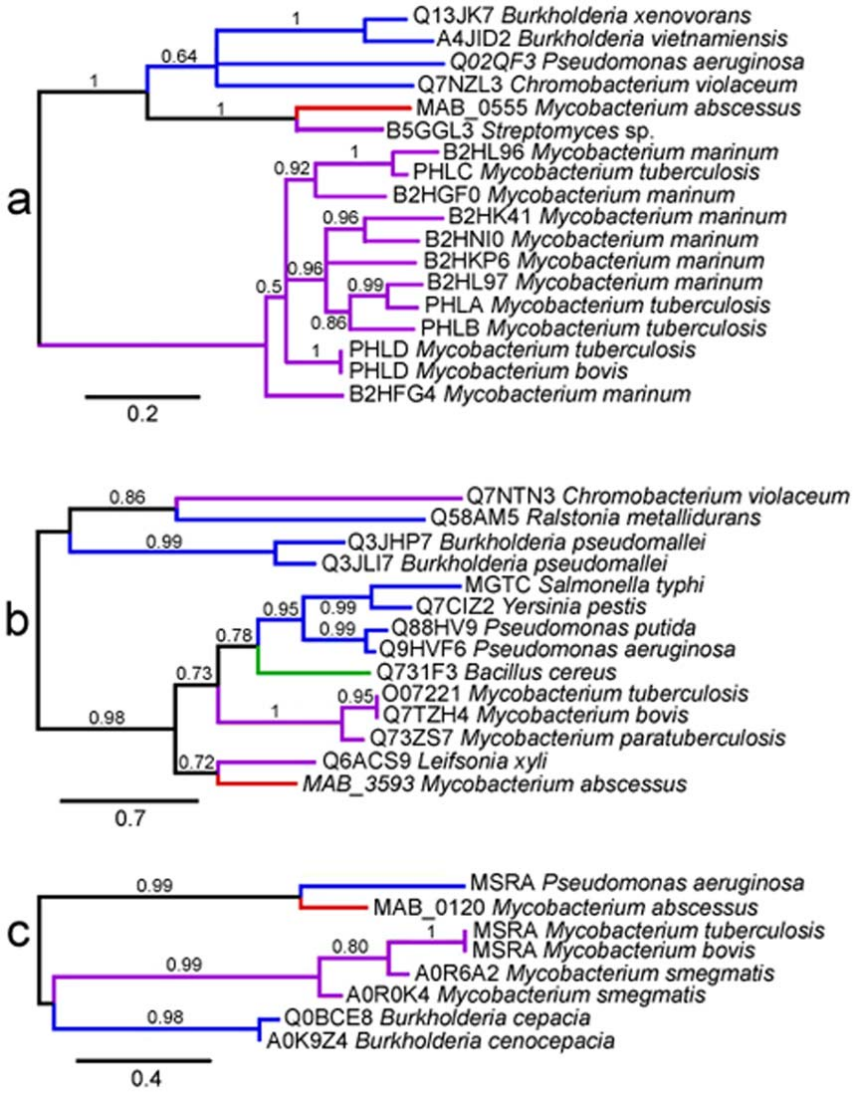
Phylogenetic trees (maximum likelihood) of PlC (a), MgtC (b) and MsrA (c) proteins. Branch supports values are indicated at the nodes. Branch colors indicate proteins from *M. abscessus* (red), Actinobacteria (purple), Proteobacteria (blue) and Firmicutes (green). Labels at the leaves show the Uniprot identifier of the proteins and the species they belong to.

Bacterial phospholipases C are key virulence factors allowing intracellular pathogens to escape phagosomal vacuoles by disrupting eukaryotic membranes [39]. *M. tuberculosis* has four phospholipase C-encoding genes: *plcABC* and *plcD* [31]. Triple (*plcABC*) and quadruple (*plcABCD*) mutants have negligible enzyme activity and attenuated virulence in mouse models, suggesting that phospholipase C activity is required for the growth of mycobacteria *in vivo* [40]. Phospholipase C activity may be particularly critical for mycobacteria infecting human hosts, as suggested by the presence of the region encompassing *plcABC* in clinical *Mycobacterium microti* isolates, but not in attenuated isolates from voles [41]. The *M. abscessus* phospholipase C closely resembles proteins from *Streptomyces* sp., *Chromobacterium violaceum* and *P. aeruginosa*. The locus containing the corresponding gene differs from those of the phospholipase C genes in *M. tuberculosis* (Fig. 4), and phylogenetic analysis is also consistent with horizontal acquisition from non mycobacterial organisms (Fig. 5).

#### MgtC

Intracellular pathogens make use of MgtC proteins to increase intracellular Mg^2+^ concentration, facilitating their survival within cells. As first demonstrated in *Salmonella*, these proteins are essential for bacterial growth within professional phagocytes, such as macrophages [42]. *M. tuberculosis* has an *mgtC* gene, the disruption of which strongly attenuates virulence in both macrophages and mice [43]. The acquisition of *mgtC* genes by HGT is common among microbes and has been associated with pathogenicity [44]. There is an *mgtC* gene in *M. abscessus*, but not in *M. smegmatis*, providing further evidence that this gene is important for the intracellular lifestyle developed by *M. abscessus*. The *M. abscessus mgtC* gene seems to have been acquired by HGT, probably from actinobacteria (Fig. 4 and 5) [44].

#### MsrA

The peptide methionine sulfoxide reductase (MsrA) of *M. tuberculosis* is thought to protect this organism against the oxidative damage caused by the reactive nitrogen intermediates produced by macrophages [45]. The *M. abscessus msrA* gene is located at another genomic site, in the immediate vicinity of *sodA* (Fig. 4). The MsrA protein of *M. abscessus* closely resembles a protein from *Rhodococcus* sp. and does not cluster with mycobacterial proteins in the phylogenetic tree for MsrA (Fig. 5), providing further evidence for horizontal acquisition from a non mycobacterial gene pool.

#### Iron uptake

Bacterial pathogens have developed a number of systems for acquiring iron, which is present in limiting concentrations in living hosts. *M. abscessus* has a four-gene cluster very similar to a locus encoding an ABC Fe(3+) transporter in *Rhodococcus* sp. This cluster also has closely related homologs in a number of major bacterial pathogens, including *Salmonella* sp. and *Burkholderia* sp. (Tables 2 and S4; Fig. 3D).

### “Non mycobacterial” factors relevant to the infection of CF patients (Tables 2 and S4; Fig. 3)

#### Phenazine biosynthesis

Phenazines are secondary metabolites with broad-spectrum antibiotic activity against bacteria, fungi and parasites, produced by *Pseudomonas* sp. and *Streptomyces* sp. Pyocyanin, the blue phenazine synthesized by *P. aeruginosa*, is a key virulence determinant *in vivo*, and is thought to be involved in the persistence of this pathogen in CF patients [46].

#### Homogentisate catabolism

The homogentisate catabolic pathway results in homogentisate being broken down into two compounds of central metabolism: fumarate and acetoacetate [47]. However, homogentisate is also a precursor for the biosynthesis of pyomelanin, a brown pigment generated through the extracellular accumulation and polymerization of homogentisate. Pyomelanin production appears to be a key step in the process by which *P. aeruginosa* adapts to the respiratory tract of CF patients, possibly facilitating iron acquisition [48].

#### Phenylacetic acid degradation

The region involved in phenylacetic acid degradation—the largest region of the *M. abscessus* genome acquired by HGT—is located downstream from the “homogentisate catabolism” region. The proteins encoded by this region constitute a complex functional unit, the phenylacetyl-CoA catabolon, which transforms various aromatic compounds (e.g., styrene, 2-phenylethylamine) into phenylacetyl-CoA, which is subsequently catabolized into TCA intermediates [49]. The five-gene cluster putatively encoding the enzymes of the ring-hydroxylating complex (MAB_0906-0910) is also present in *B. cepacia* and the homolog of MAB_0910 is essential for *B. cepacia* survival in a rat model of chronic lung infection [50].

#### Cysteine desulfurases and the “Dnd phenotype”

The *M. abscessus* genome encodes a large number of cysteine desulfurases (Table S3). One is part of a locus closely resembling the *S. lividans dndA-E* locus (Tables 2 and S4; Fig. 3), which is involved in the Dnd (DNA degradation) phenotype observed *in vitro* during DNA extraction [51]. A recent pulsed-field gel electrophoresis study showed that *M. abscessus* strains with a Dnd phenotype belonged to a small number of closely related clones playing a major role in human disease [52]. The sputum of CF patients is extremely rich in DNA, which may constitute an important source of nutrients for *dnd*-positive strains.

### Resistance to antimicrobial compounds

*M. abscessus* has achieved notoriety as one of the most drug-resistant mycobacterial species [1]. Much of this multidrug resistance may result from weak permeability of the cell wall, but analysis of the *M. abscessus* genome has also revealed the presence of many potential drug resistance determinants. The hydrolytic or drug-modifying enzymes present in this species include an Ambler class A beta-lactamase, a rifampin ADP-ribosyl transferase, an aminoglycoside 2′-N-acetyltransferase and at least 12 homologs of aminoglycoside phosphotransferases. We also found four homologs of monooxygenases potentially involved in resistance to rifampin and tetracyclines, two FolP homologs conferring resistance to cotrimoxazole, one homolog of UDP-N-acetylglucosamine 1-carboxyvinyltransferase MurA conferring resistance to fosfomycin, and two homologs of 23S rRNA methylases conferring resistance to macrolides, including the *erm* gene product (MAB_2297) recently shown to be involved in inducible macrolide resistance in *M. abscessus* [53]. Most of these drug resistance determinants are mycobacterial, with the notable exception of the Ambler class A beta-lactamase (MAB_2875), which closely resembles beta-lactamases from the gram-negative bacteria *Pseudomonas luteola* and *Serratia fonticola* (not shown). The genome of *M. abscessus* also encodes many proteins potentially involved in drug-efflux systems, including members of the major facilitator family, ABC transporters and MmpL proteins. Finally, the presence of a single rRNA operon favors the occurrence of dominant mutations conferring resistance to aminoglycosides and macrolides [54].

We identified three putative *ars* operons scattered over the chromosome of *M. abscessus* [55]. *M. abscessus* is therefore likely to be resistant to high concentrations of arsenic [56]. Finally, due to the presence of *merB* within the *mer* operon, the 23 kb plasmid probably confers resistance to a wide range of organomercury compounds [57].

## Discussion

### Deciphering the ecology and biology of *M. abscessus*

The genetic information contained in the genome of *M. abscessus* tells us a great deal about the lifestyle of this microorganism in natural conditions. The presence of a large number of genes and operons involved in resistance to arsenic or encoding cysteine desulferases is clearly a hallmark of an environmental organism living in soil or aquatic environments. However, *M. abscessus* also contains a whole series of genes known to be involved in intracellular survival (e.g., *mgtC*, *msrA*, *plc*), and is well-equipped to obtain energy from the degradation of eukaryotic host-derived lipids (numerous lipase-encoding genes), as observed for mycobacteria adapted to an intracellular lifestyle [58]. The low level of metabolic versatility (e.g., far fewer ABC transporters or two-component sensor histidine kinases than *M. smegmatis*) suggests that this bacterium tends to specialize in intracellular parasitism.

The most plausible hypothesis is that *M. abscessus* has evolved to escape predators, such as free-living amebas [8] sharing the same ecosystem. Soil-dwelling amebas are known to be most abundant at plant-soil interfaces, because these interfaces support the growth of various plant parasites, including bacteria, on which amebas feed [59]. Consistent with this hypothesis, the genome of *M. abscessus* encodes a particularly large number of salicylate hydroxylases, enabling this bacterium to resist the salicylic acid-mediated defense mechanisms of plants [60]. This suggests that *M. abscsessus* lives in close contact with plants and therefore has to deal with amebas. This hypothesis may explain an extraordinary paradox in the epidemiology of *M. abscessus*: despite all the evidence to suggest that *M. abscessus* lives in soil and water—our own genomic data and the large number of epidemics linked to the direct or indirect use of non sterile water—this bacterium is detected much less frequently in such environments than other closely related RGM, such as *M. chelonae* [61].

We analyzed an S phenotype strain. A major challenge for the future will be to determine the role of S↔R switches in the natural lifecycle of *M. abscessus* (controlling whether this bacterium grows in the form of a biofilm) and its interaction with its hosts, including humans (modulation of the host response). GPL may be required for biofilm establishment or for escape from amebas in aquatic environments [62], but seems to hinder the development of infection, probably by acting as a target of the specific immune response of the host [22]. We recently reported the *in vivo* isolation of an R variant from the type strain CIP 104536T [22]. Transcriptomic studies are currently underway to determine the mechanisms responsible for the loss of GPL production in this R variant and the associated events potentially accounting for its “hypervirulence” in mice. The data obtained should make it possible to identify the external signals involving in triggering the switching process.

### Evolutionary mechanisms

This study highlights the major role of horizontal gene transfers in the evolution of RGM. It is hardly surprising that this evolutionary mechanism, which has also been described in SGM [63], [64], is particularly important in RGM, and that it involves a reservoir of genes from different bacteria with a high G+C content widely present in soil or water, such as *Streptomyces* sp., *Rhodococcus* sp. and pseudomonads. RGM come into contact with many other bacteria in the environment—often as part of a biofilm [65] —and they may exchange genetic material with these other bacteria [66]. Mycobacteriophages—or other bacteriophages with a wide host spectrum—may play a key role in such transfers, as they display extensive mosaicism, combining viral and bacterial genes in a vast gene pool [32]. Such a role in gene transfer is consistent with the presence of a full-length prophage sequence containing non mycobacterial genes in the *M. abscessus* genome. However, the presence of this prophage sequence does not exclude a role for other genetic vectors, such as plasmids, which are frequently found free or integrated into the genome within RGM [67].

The demonstration that pathogenicity genes of non mycobacterial origin are present in *M. abscessus* raises questions about the timing of their acquisition. The fact that the closely related species *M. chelonae* is also pathogenic in humans—an exceptional feature among RGM—strongly suggests that many of these genes were acquired before the separation of these two species. We are currently carrying out a comparative genomics study of *M. abscessus* and *M. chelonae* (http://www.genoscope.cns.fr/spip/Mycobacterium-chelonae-and.html), which should make it possible to confirm or to infirm this hypothesis. We have also recently made use of the genome sequence of *M. abscessus* to develop a multilocus sequence typing (MLST) approach. Our preliminary analyses on more than a hundred *M. abscessus* (*sensu lato*) strains suggest that there are three highly homogeneous groups, corresponding to the three previously described species (*M. abscessus sensu stricto*, *M. massiliense*, *M. bolletii*), with less than 1% divergence within groups and around 2% divergence between groups. The species of *M. abscessus sensu lato* therefore seem to have emerged relatively recently. However, it should be stressed that most of the strains of *M. abscessus* available from collections were isolated recently and mostly in a clinical context. Indeed, as stated above, *M. abscessus* is only very rarely isolated from the environment. There may therefore be a bias in the results, because we cannot rule out the possibility that strains capable of infecting humans constitute an unusual subpopulation.

One of the findings of this study was entirely unexpected: the presence of a mercury resistance plasmid almost identical to the pMM23 from the *M. marinum* strain recently sequenced by the team of Stinear (strain ATCC BAA-535) [30]. The pMM23 plasmid discovered in this strain, isolated from a patient in 1992 (Moffett Hospital, San Francisco), is exceptional in *M. marinum*, as none of the more than 40 other isolates of this species studied by the team of Stinear has been found to carry this plasmid [30]. The presence of this plasmid in *M. abscessus* is, thus, particularly interesting, as it demonstrates that exchanges may occur between *M. marinum*, a SGM, and *M. abscessus*, a RGM, either directly or via another organism, probably a mycobacterium. It also suggests that *M. abscessus* and *M. marinum* may live in the same ecosystems and may be transmitted to humans by similar mechanisms. Future work should determine the prevalence of this plasmid in *M. abscessus* and should assess whether this plasmid constitutes a useful marker (e.g., for epidemicity).

We were also surprised by the very low frequency of IS in the genome of the strain of *M. abscessus* that we sequenced, much lower than usually found in mycobacteria. Confirmation of this result is required, with a representative panel of isolates. If confirmed, this characteristic would have a major impact on the plasticity of the genome of *M. abscessus*. As elegantly demonstrated in *Escherichia coli*, reducing the number of IS elements renders bacterial genomes more stable, with a greater capacity for acquiring foreign DNA [68].

### Key factors shared with other major CF pathogens

This study provides new insight into the emergence of *M. abscessus* as a pathogen in CF patients. We were surprised to discover that the largest tranferred regions detected in *M. abscessus* contained genes involved in the metabolism of aromatic compounds. Such systems are characteristic of pseudomonads in general, and of two major CF pathogens, *P. aeruginosa* and *B. cepacia*, in particular [49]. This implies that *M. abscessus* is able to live in the same ecosystems as *P. aeruginosa* and *B. cepacia*, with patients becoming infected from the same microbial reservoir. Another, not necessarily exclusive possibility is that these metabolic characteristics provide a selective advantage in CF patients, due either to their illness or the treatments increasingly used over recent years, such as aerosolized drug administration [69]. According to this hypothesis, *M. abscessus* may benefit from factors promoting its extracellular development and its implantation in the bronchial tract, before going on to cause deeper infection of the pulmonary parenchyma and ganglions.

Conversely, several *M. abscessus* factors typical of intracellular parasites are also present in *P. aeruginosa* and *B. cepacia*, the most notable examples being phospholipase C and the MgtC protein [70], [71]. Both *P. aeruginosa* and *B. cepacia* produce two phospholipases C and two MgtC proteins [70], [71]. An MgtC-like protein is also found in *Aspergillus fumigatus*—the main pathogenic fungus in CF patients—but not in closely related nonpathogenic species such as *Aspergillus nidulans*. Pseudomonads and other related organisms infecting CF patients have previously been considered to be exclusively “extracellular” pathogens. Our data raise questions about the interaction of these organisms with macrophages or other monocyte-derived cells in CF patients. This is consistent with the finding that the production of MgtC is required for the survival of *Burkholderia cenocepacia*–the main *B. cepacia* complex pathogen infecting CF patients–within macrophages [72], [73].

A recent analysis of the genomes of various CF and non-CF *P. aeruginosa* isolates revealed mosaic structures, consisting of a conserved core component interrupted by strain-specific genomic islands acquired by HGT, which seem to provide CF isolates with specific metabolic pathways involved in infection [74]. The identification of multiple episodes of HGT in *M. abscessus* strongly suggests that a similar evolutionary trend occurs within RGM. Along the same lines as the studies carried out in *P. aeruginosa* by the team of Lowry [74], comparative genomic studies of CF and non-CF *M. abscessus* isolates could prove particularly fruitful for elucidating the tropism of certain organisms for the respiratory tract of CF patients, opening up promising new possibilities for the control of microbial infections in CF patients.

## Materials and Methods

We sequenced *M. abscessus* (*sensu stricto*) CIP 104536T ( = ATCC 19977T), using a whole-genome shotgun strategy (EMBL accession numbers: CU458896, chromosome; CU458745, plasmid). This strain is of the S phenotype, and can switch *in vivo* to an R phenotype [22]. Mycobacteria were grown in Middlebrook 7H9 broth supplemented with Tween 80. *M. abscessus* DNA, prepared using standard methods, was manipulated in the presence of 50 µM thiourea (DNA in solution) or by replacing Tris buffer by HEPES at the same molarity (DNA in plugs), to prevent Tris-dependent DNA degradation [39]. We constructed three genomic libraries (inserts of 3–4, 8–10 and ∼20 kb, respectively) and generated ∼80,000 sequences (50,000, 20,000 and 10,000 sequences, respectively, giving 11-fold coverage). Putative protein-coding sequences were predicted by SHOW (http://migale.jouy.inra.fr/outils/select_mig_outils_zpt), tRNA genes by tRNAscan, and rRNA genes by RNAmmer [75], [76]. Sequences were analyzed with the BIOFACET package and the BLAST software suite [77], [78]. General features, such as G+C content (%), were assessed with ARTEMIS software [79]. The origin of replication was identified with ORILOC [80]. The circular representations of chromosome and plasmid were generated with DNAPlotter (http://www.sanger.ac.uk/Software/Artemis/circular). The *M. abscessus* full-length prophage was drawn with BugView (http://www.gla.ac.uk/~dpl1n/BugView/index.html). Whole genome dotplot comparison of *M. abscessus versus M. smegmatis* was drawn with Gepard (http://mips.gsf.de/services/analysis/gepard). CLUSTER-C was used to cluster genes into paralogous families [81]. Alien Hunter was used to screen the genome for regions with “atypical” sequence content [82]. Transfers of blocks of genes from non mycobacterial organisms were identified as follows. We first identified CDS more similar to proteins from non mycobacterial organisms than to mycobacterial proteins (no mycobacterial protein among the 50 best hits). We then used GeneTeam, with a delta value of 3 and visual inspection to search for areas of synteny with relevant non mycobacterial organisms [83]. Only clusters with at least 3 syntenic genes not found in other sequenced mycobacteria were retained. Phylogenetic analyses were carried out with the “Phylogeny.fr” web server (http://www.phylogeny.fr), using Muscle for multiple alignment and GBlocks for alignment curation, and constructing the phylogenetic trees with PhyML [84], [85]. Branch supports were calculated with the approximate likelihood ratio test [86]. Distributions of *M. abscessus* and *M. smegmatis* proteins, according to the Kegg classification, were compared using chi-squared tests with continuity correction. To account for multiple testing, p-values were corrected according to Hochberg's method. Differences were considered as statistically significant if corrected p-values were <0.05.

## Supporting Information

Figure S1 Whole genome dotplot comparison of M. abscessus (horizontal axis) versus M. smegmatis.
(0.08 MB TIF)

Table S1 M. abscessus prophage-like elements
(0.03 MB DOC)

Table S2 M. abscessus and M. smegmatis proteins involved in metabolism, according to the Kegg classification
(0.04 MB DOC)

Table S3 A selection of paralogous families
(0.06 MB DOC)

Table S4 Proteins encoded in the 17 horizontally acquired gene clusters, and their syntenic non mycobacterial homologs
(0.06 MB DOC)

Table S5 (0.06 MB DOC)
