# Supplementary figures and images for: Non Mycobacterial Virulence Genes in the Genome of the Emerging Pathogen Mycobacterium abscessus
Source: PLoS One. 2009 Jun 19;4(6):e5660. doi: 10.1371/journal.pone.0005660 (PMC2694998; doi:10.1371/journal.pone.0005660)

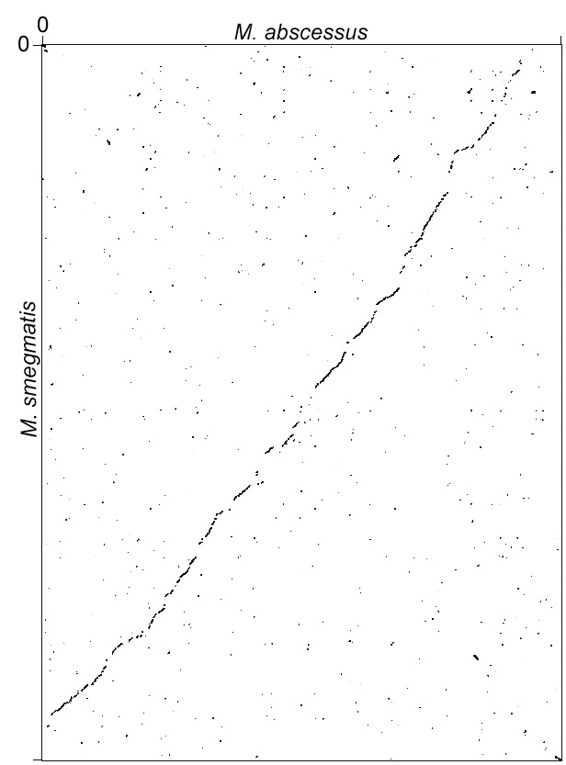

Supplement: Figure S1 — Whole genome dotplot comparison of M. abscessus (horizontal axis) versus M. smegmatis. (0.08 MB TIF) [file pone.0005660.s001.tif]
