# Supplementary material for: Non Mycobacterial Virulence Genes in the Genome of the Emerging Pathogen Mycobacterium abscessus
Source: PLoS One. 2009 Jun 19;4(6):e5660. doi: 10.1371/journal.pone.0005660 (PMC2694998; doi:10.1371/journal.pone.0005660)
Supplement: Table S1 — M. abscessus prophage-like elements (0.03 MB DOC) [file pone.0005660.s002.doc]

Table S1. *M. abscessus* prophage-like elements

| Coordinates  (bp) | Length  (bp) | *M. abscessus* CDS | Comments |
| --- | --- | --- | --- |
| 233621 - 247981 | 14361 | MAB_0221c-0242 | Integrated near Arg- and Ser tRNA sequences. Presence of genes encoding an integrase (MAB_0221c) and a putative DNA-binding protein (MAB_0222c). Other genes encode hypothetical proteins and a putative methyltransferase. |
| 770916.-.778753 | 7838 | MAB_0772c-0783c | Probable prophage remnant. Integrated near Pro-, Phe-, Asp-, Glu- and Lys tRNA sequences. Presence of a gene encoding a bacteriophage protein (MAB_0783c). Other genes encode hypothetical proteins. |
| 4909957.-.4959626 | 49670 | MAB_4802-4848c | Possible prophage remnant. Harbors genes encoding one resolvase (MAB_4811c), one putative helicase (MAB_4847c) and two bacteriophage proteins (MAB_4807, MAB_4822). Presence of a putative *ars* operon. |
