# Supplementary material for: Non Mycobacterial Virulence Genes in the Genome of the Emerging Pathogen Mycobacterium abscessus
Source: PLoS One. 2009 Jun 19;4(6):e5660. doi: 10.1371/journal.pone.0005660 (PMC2694998; doi:10.1371/journal.pone.0005660)
Supplement: Table S2 — M. abscessus and M. smegmatis proteins involved in metabolism, according to the Kegg classification (0.04 MB DOC) [file pone.0005660.s003.doc]

Table S2. *M. abscessus* and *M. smegmatis* proteins involved in metabolism, according to the Kegg classification

| Kegg classification | *M. abscessus* proteins ^(a)^ | | *M. smegmatis* proteins ^(a)^ | |
| --- | --- | --- | --- | --- |
|  | No. | % | No. | % |
| Carbohydrate metabolism | 363 | 35.9 | 700 | 38.6 |
| Xenobiotics biodegradation and metabolism | 222 | 21.9 | 692 | 38.1 |
| Glycan biosynthesis and metabolism | 20 | 1.98 | 33 | 1.8 |
| Nucleotide biosynthesis | 107 | 10.6 | 200 | 11.0 |
| Biosynthesis of secondary metabolites | 57 | 5.6 | 161 | 8.9 |
| Energy metabolism | 111 | 11.0 | 256 | 14.1 |
| Biosynthesis of polyketides and NRPs | 11 | 1.1 | 17 | 0.9 |
| Metabolism of other amino acids | 98 | 9.7 | 164 | 9.0 |
| Lipid metabolism | 175 | 17.3 | 364 | 20.1 |
| Amino acid metabolism | 439 | 43.4 | 867 | 47.8 |
| Metabolism of cofactors and vitamins | 144 | 14.2 | 277 | 15.3 |

(a) Total no. of proteins classified: *M. abscessus*, 1012; *M. smegmatis*, 1814.

Abbreviations: NRPs, nonribosomal peptides.
