# Supplementary material for: Non Mycobacterial Virulence Genes in the Genome of the Emerging Pathogen Mycobacterium abscessus
Source: PLoS One. 2009 Jun 19;4(6):e5660. doi: 10.1371/journal.pone.0005660 (PMC2694998; doi:10.1371/journal.pone.0005660)
Supplement: Table S3 — A selection of paralogous families (0.06 MB DOC) [file pone.0005660.s004.doc]

Table S3. A selection of paralogous families

in *M. abscessus* and *M. smegmatis*

(a) Polyketide biosynthesis.

| Family | Occurrence | |
| --- | --- | --- |
|  | Mabs | Msmeg |
| ABC transporter, ATP-binding protein | 56 | 121 |
| Acyl-CoA dehydrogenase (fadE) | 46 | 83 |
| Aldehyde dehydrogenase | 23 | 41 |
| Aminotransferase (all types) | 42 | 46 |
| Arsenate reductase ArsC | 6 | 3 |
| b-ketoacyl- [ACP] synthase | 7 | 3 |
| Carbonic anhydrase (cytosol) | 5 | 2 |
| Catalase | 4 | 6 |
| Cation-transporting ATPase | 5 | 6 |
| Cutinase | 6 | 10 |
| Cyclopropane fatty acid synthase | 3 | 8 |
| Cysteine desulfurase | 8 | 5 |
| Cytochrome P450 | 25 | 42 |
| ECF s factor | 11 | 17 |
| Enoyl-CoA hydratase/isomerase | 26 | 52 |
| Fibronectin-binding protein | 4 | 6 |
| Glutamine synthetase | 2 | 10 |
| Glycosyltransferase | 21 | 28 |
| Lipase/esterase/monooxygenase | 62 | 37 |
| Lipoprotein LpqH precursor | 4 | 2 |
| MbtH protein | 3 | 2 |
| MCE protein | 44 | 34 |
| Membrane protein, MmpL | 31 | 16 |
| Molybdenum cofactor biosynthesis | 4 | 4 |
| O-methyltransferase Omt^(a)^ | 12 | 7 |
| Oxidoreductase, aldo-keto reductase | 3 | 14 |
| PE and PPE proteins | 9^(b)^ | 2^(b)^ |
| Salicylate hydroxylase | 5 | 3 |
| Serine/threonine protein kinase | 8 | 19 |
| Short-chain dehydrogenase/reductase | 80 | 160 |
| Superoxide dismutase | 3 | 3 |
| Transcriptional regulator, GntR family | 16 | 46 |
| Transcriptional regulator, IclR family | 5 | 15 |
| Transcriptional regulator, LuxR family | 9 | 27 |
| Transcriptional regulator, LysR family | 19 | 37 |
| Transcriptional regulator, TetR family | 115 | 130 |
| Two-component sensor histidine kinase | 16 | 28 |
| YrbE protein | 12 | 11 |

(b) Only proteins with N-terminal PE or PPE motifs were included.

Abbreviations: Mabs, *M. abscessus;* Msmeg, *M. smegmatis*
