# Supplementary material for: Non Mycobacterial Virulence Genes in the Genome of the Emerging Pathogen Mycobacterium abscessus
Source: PLoS One. 2009 Jun 19;4(6):e5660. doi: 10.1371/journal.pone.0005660 (PMC2694998; doi:10.1371/journal.pone.0005660)
Supplement: Table S4 — Proteins encoded in the 17 horizontally acquired gene clusters, and their syntenic non mycobacterial homologs (0.06 MB DOC) [file pone.0005660.s005.doc]

Table S4. Proteins encoded in the 17 horizontally acquired gene clusters, and their syntenic non mycobacterial homologs

| Cluster no. | Syntenic organism | Mabs proteins | Homolog in the synteny ^(a)^ | Best mycobacterial hit ^(c) (d)^ |
| --- | --- | --- | --- | --- |
| 1 | *Streptomyces* sp*.* MG1 | MAB_0295  MAB_0296  MAB_0297  MAB_0298 | B4V5P4 ^(b)^ =phenazine biosynthesis protein PhzC (e-118) ^(c)^  B4V5P3 = 2,3-dihydroxybenzoate-2,3 dehydrogenase (8e-55)  B4V5P2 =phenazine biosynthesis protein PhzD (3e-68)  B4V5P1 =phenazine biosynthesis protein PhzE (0.0) | *Mycobacterium smegmatis* (7e-71) [210]  No  No  *Mycobacterium marinum* (2e-09) [408] |
| 2 | *Nocardia farcinica* | MAB_0300c  MAB_0301  MAB_0302  MAB_0303 | Q5YP17=putative transcriptional regulator (1e-39)  Q5YP18=putative carboxyesterase (3e-84)  Q5YP19=putative monooxygenase (e-103)  Q5YP20=putative dioxygenase (1e-97) | *Mycobacterium smegmatis* (2e-08) [17]  *Mycobacterium smegmatis* (3e-50) [4]  *Mycobacterium gilvum* (3e-14) [27]  No |
| 3 | *Rhodococcus* sp. | MAB_0888c  MAB_0889c  MAB_0890c  MAB_0891c | Q0SEC1=probable fumarylacetoacetase (e-157)  Q0SEC0=hypothetical protein (7e-43)  Q0SEB9=homogentisate 1,2-dioxygenase (0.0)  Q0SEB8=probable transcriptional regulator, MarR (2e-53) | No  No  *Mycobacterium smegmatis* (7e-31) [147]  No |
| 4 | *Nocardia farcinica* | MAB_0899c  MAB_0900c  MAB_0901c  MAB_0902  MAB_0903  MAB_0904  MAB_0905  MAB_0906  MAB_0907  MAB_0908  MAB_0909  MAB_0910  MAB_0911 | Q5YXU1=hypothetical protein (3e-22)  Q5YXU0=putative dehydrogenase (0.0)  Q5YXT9=putative transcriptional regulator (9e-54)  Q5YXT8=putative acyl-CoA thiolase (e-139)  Q5YXT7=putative enoyl-CoA hydratase/isomerase (6e-73)  Q5YXT6=putative 3-hydroxyacyl-CoA dehydrogenase (2e-86)  Q5YXT5=putative enoyl-CoA hydratase/isomerase (5e-62)  Q5YXT4=putative phenylacetic acid degradation protein (e-145)  Q5YXT3=putative phenylacetic acid degradation protein (6e-42)  Q5YXT2=putative phenylacetic acid degradation protein (4e-97)  Q5YXT1=putative phenylacetic acid degradation protein (3e-45)  Q5YXT0=putative phenylacetic acid degradation NADH oxidoreductase (e-135)  Q5YXS9=putative phenylacetate-CoA synthetase (0.0) | *Mycobacterium* sp. (6e-29) [2]  *Mycobacterium smegmatis* (4e-19] [192]  No  No  *Mycobacterium gilvum* (2e-24) [72]  *Mycobacterium smegmatis* (4e-65) [9]  *Mycobacterium gilvum* (8e-25) [225]  No  No  No  No  *Mycobacterium bovis* ((E-70) [70]  No |
| 5 | *Rhodococcus* sp. | MAB_1014c  MAB_1015c  MAB_1016c  MAB_1017c  MAB_1018c  MAB_1019c | Q0S4M6=hypothetical protein (1e-90)  Q0S4M5=hypothetical protein (1e-63)  Q0S4M1=hypothetical protein (e-130)  Q0S4M0=probable O-antigen transporter, MOP superfamily (2e-93)  Q0S4L9=possible glycosyltransferase (e-113)  Q0S4L8=probable glycosyltransferase (3e-99) | *Mycobacterium smegmatis* (2e-77) [2]  *Mycobacterium smegmatis* (2e-38) [2]  No  No  No  *Mycobacterium gilvum* (4e-16) [120] |
| 6 | *Streptomyces lividans* | MAB_1093c  MAB_1094  MAB_1095  MAB_1096  MAB_1097  MAB_1098 | Q460H9=putative pyridoxal phosphate-dependent L-cysteine desulfurase (1e-95)  Q460H8=putative ATPase (e-116)  Q460H7=putative sulfurtransferase (e-179)  Q460H6=putative ATPase (1e-84)  Q460H5=putative phosphoribosylaminoimidazole carboxylase synthetase (4e-27)  Q460I0=putative SAM-dependent methyltransferase (2e-81) | No  No  No  No  No  No |
| 7 | *Rhodococcus* sp. (strain RHA1) | MAB_1501  MAB_1502  MAB_1503  MAB_1504 | Q0S261=possible ABC Fe(3+) transporter, permease component (e-106)  Q0S262=probable ABC Fe(3+) transporter, ATP-binding component (e-119)  Q0S263=ABC transporter, permease component (5e-94)  Q0S264=ABC transporter, permease component (7e-62) | No  *Mycobacterium vanbaalenii* (2e-52) [180]  *Mycobacterium marinum* (8e-12) [212]  No |
| 8 | *Rhodococcus* sp. | MAB_1720  MAB_1721  MAB_1722 | Q0S6K3=response regulator, two-component system (3e-68)  Q0S6K2=sensor kinase, two-component system (e-100)  Q0S6K1=hypothetical protein (1e-80) | *Mycobacterium vanbaalenii* (5e-37) [71]  *Mycobacterium* sp. strain JLS (3e-34) [48]  No |
| 9 | *Pseudomonas putida* (strain F1) | MAB_2027  MAB_2028  MAB_2029  MAB_2030  MAB_2031  MAB_2032 | A5W4P6=putative uncharacterized protein (2e-10)  A5W4P5=beta-ketoacyl synthase-like protein (8e-75)  A5W4P4=putative uncharacterized protein (5e-08)  A5W4P3=beta-ketoacyl synthase-like protein (2e-98)  A5W4P2=beta-ketoacyl synthase-like protein (2e-32)  A5W4P0=short-chain dehydrogenase/reductase (3e-51) | No  *Mycobacterium gilvum* (8e-49) [181]  No  No  No  No |
| 10 | *Burkholderia cepacia* complex | MAB_2251  MAB_2252  MAB_2253 | A9AQQ0=Helix-turn-helix protein (2e-13)  A9AQQ1=Hypothetical protein (4e-31)  A9AQQ2=putative methyltransferase (2e-81) | No  No  *Mycobacterium smegmatis* (1e-29) [73] |
| 11 | *Myxococcus xanthus* | MAB_2255  MAB_2256  MAB_2257 | Q1D6A2=non-ribosomal peptide synthase (0.0)  Q1D6A6=polyketide synthase type I (0.0)  Q1D6A5=polyketide synthase type I (0.0) | *Mycobacterium paratuberculosis* (e-136) [484]  *Mycobacterium gilvum* (0.0) [74]  *Mycobacterium tuberculosis* ((e-179) [74] |
| 12 | *Streptomyces ambofaciens* | MAB_2257  MAB_2258 | A3KI34=putative polyketide synthase (0.0)  A3KI35=putative peptide synthetase (e-175) | *Mycobacterium tuberculosis* ((e-179) [74]  *Mycobacterium vanbaalenii* (4e-53) [466] |
| 13 | *Streptomyces coelicolor* | MAB_2278  MAB_2279  MAB_2280  MAB_2281  MAB_2282  MAB_2283  MAB_2284  MAB_2286 | Q9K3F5=putative oxidoreductase (3e-74)  Q9K3F7=putative aminotransferase (e-107)  Q9K3F8=hypothetical protein (3e-21)  Q9K3F3=putative integral membrane protein (1e-36)  Q9K3F9=putative oxidoreductase (6e-22)  Q9K3G0=putative oxidoreductase (8e-39)  Q9K3G5=putative halogenase (0.0)  Q9K3F4=hypothetical protein (1e-79) | No  *Mycobacterium smegmatis* (5e-48) [148]  No  No  *Mycobacterium vanbaalenii* (9e-15) [4]  *Mycobacterium smegmatis* (4e-28) [118]  No  No |
| 14 | *Bacillus pumilus* | MAB_2610  MAB_2611  MAB_2612  MAB_2613 | B4AMJ6=pyridoxal-phosphate-dependent aminotransferase)(e-107)  B4AMJ5=HAD-superfamily hydrolase (3e-45)  B4AMJ4=oxidoreductase Gfo/Idh/MOCA (4e-64)  B4AMJ3=MFS family major facilitator transporter (1e-21) | *Mycobacterium avium* complex (2e-54) [36]  No  No  No |
| 15 | *Nocardia farcinica* | MAB_3112  MAB_3113  MAB_3114  MAB_3115 | Q5YN04=hypothetical protein (2e-21)  Q5YN03=hypothetical protein (1e-27)  Q5YN02=hypothetical protein (e-105)  Q5YN01=hypothetical protein (8e-20) | No  No  No  No |
| 16 | *Streptomyces antibioticus* | MAB_3569c  MAB_3571c  MAB_3572c  MAB_3573c  MAB_3574c | Q0R4L5=ChlD4 (2e-34)  Q0R4L6=ChlD3 (8e-33)  Q0R4L7=ChlD2 (5e-07)  Q0R4L8=ChlD1 (5e-91)  Q0R4L9=ChlM (4e-78) | No  No  No  *Mycobacterium avium* complex (7e-47) [38]  *Mycobacterium avium* complex (2e-15) [41] |
| 17 | *Rhodococcus* sp. | MAB_3621c  MAB_3622c  MAB_3623 | Q0SA51=taurine dioxygenase (e-135)  Q0SA50=quinone oxidoreductase (e-127)  Q0SA49=probable transcriptional regulator, AraC family (e-127) | *Mycobacterium smegmatis* (3e-50) [75]  *Mycobacterium smegmatis* (3e-71) [190]  *Mycobacterium bovis* (4e-21) [55] |

(a) Only the best syntenies are shown (see also Table 2).

(b) Entry name in Swiss-Prot/TrEMBL database.

(c) As indicated in Uniprot; ()=E-values.

(d) The mycobacterial protein with the best E-value among the 500 best hits is shown; [], rank among the 500 best hits; "no" indicates the absence of mycobacterial proteins among the 500 best hits.

Abbreviation: Mabs, *M. abscessus*.
