# Supplementary material for: Non Mycobacterial Virulence Genes in the Genome of the Emerging Pathogen Mycobacterium abscessus
Source: PLoS One. 2009 Jun 19;4(6):e5660. doi: 10.1371/journal.pone.0005660 (PMC2694998; doi:10.1371/journal.pone.0005660)
Supplement: Table S5 — (0.06 MB DOC) [file pone.0005660.s006.doc]

Table S5. “Mycobacterial” homologs of *M. tuberculosis* virulence factors in *M. abscessus* and *M. smegmatis*^(a)^

| **Name** | **Mtb n°** | **Main function (reference)** | **Mabs n°** | **% id.** | **Msmeg n°** | **% id.** |
| --- | --- | --- | --- | --- | --- | --- |
| AhpC | Rv2428 | Alkyl hydroperoxide reductase C. Thought to detoxify the hydroxyperoxides produced by macrophages | MAB_4408c | 83% | Msmeg_4891 | 82% |
| AhpD | Rv2429 | Alkyl hydroperoxide reductase D. Involved in detoxification of hydroxyperoxides. Also regenerates oxidized AhpC | MAB_4407c | 67% | Msmeg_4890 | 64% |
| DlaT | Rv2215 | Dihydrolipoamide acyltransferase. Regenerates oxidized AhpD | MAB_1945c | 76% | Msmeg_4283 | 78% |
| DosR | Rv3133c | Two-component response regulator (global response to anoxic conditions) | MAB_3891c | 71% | Msmeg_5244 | 84% |
| Erp | Rv3810 | Surface protein involved in virulence | MAB_0169c | 45% | Msmeg_6405 | 54% |
| FbpA | Rv3804 | Mycolyl-transferase enzyme with fibronectin-binding properties | MAB_0176 | 63% | Msmeg_6398 | 68% |
| GlnA1 | Rv2220 | Nitrogen metabolism and synthesis of poly-L-glutamate-glutamine cell wall component | MAB_1933c | 85% | Msmeg_4290 | 84% |
| HbhA | Rv0475 | Heparin-binding hemagglutinin protein | MAB_4083c | 59% | Msmeg_0919 | 64% |
| HspX | Rv2031 | Chaperone-like protein induced under anoxic conditions (DosR-dependent response) | MAB_4402 | 38% | Msmeg_3932 | 60% |
| Icl | Rv0467 | Isocitrate lyase. Plays a key role in the survival of mycobacteria within activated macrophages | MAB_4095c | 83% | Msmeg_0911 | 92% |
| IdeR | Rv2711 | Major mycobacterial regulator of iron metabolism genes | MAB_3029 | 81% | Msmeg_2750 | 86% |
| IipA | Rv1477 | Protein involved in the invasion and intracellular persistance of mycobacteria | MAB_2728c^(b)^ | 49% | Msmeg_3145^(b)^ | 59% |
| IrtA | Rv1348 | ABC transporter required for the growth of *M. tuberculosis* in iron-deficient conditions | MAB_2262c^(c)^ | 62% | Msmeg_6554^(c)^ | 71% |
| KatG | Rv1908 | Catalase peroxidase. Is thought to contribute to degrade ROI produced by macrophages | MAB_2470c | 72% | Msmeg_6384 | 71% |
| LpqH | Rv3763 | Immunodominant antigen. A surface-exposed glycoprotein also thought to modulate the host cytokine response | MAB_0885c^(d)^ | 53% | Msmeg_6310 | 50% |
| MbtB | Rv2383 | Mycobactin synthesis (formation of an amide bond between salicylate and serine) | MAB_2124 | 50% | Msmeg_4515 | 62% |
| MgtC | Rv1811 | Mg^2+^ transporter required for bacteria to grow within macrophages | No^(e)^ | NA | No | NA |
| MsrA | Rv0137c | Methionine sulfoxide reductase. May protect intracellular bacteria against oxidative damage from RNI | No^(e)^ | NA | Msmeg_4417 | 79% |
| NarG | Rv1161 | Subunit of the nitrate reductase. May be required in anaerobic and oxygen-poor environments | No^(f)^ | NA | Msmeg_5140 | 79% |
| OmpA | Rv0899 | A porin-like protein induced by low pH and during growth in macrophages | No | NA | No | NA |
| PhoP | Rv0757c | Two-component response regulator sensing Mg^2+^ starvation | MAB_0673 | 86% | Msmeg_5872 | 93% |
| PlcD^(g)^ | Rv1755c | Phospholipase C | No^(e)^ | NA | No | NA |
| PrrA | Rv0903c | Response regulator. Upregulated during infection of human macrophages | MAB_0956c | 91% | Msmeg_5662 | 95% |
| RpfA | Rv0867c | Autocrine growth factor required to resuscitate dormant bacterial cells | MAB_0869c^(h)^ | 44% | Msmeg_5700^(h)^ | 50% |
| SigC^(i)^ | Rv2069 | ECF sigma factor involved in stress response and virulence | MAB_3428c | 62% | No | NA |
| SodA | Rv3846 | Iron-coordinated superoxide dismutase.Thought to be involved in detoxifying the ROI produced by macrophages | MAB_0118c | 81% | Msmeg_6327 | 80% |
| SodC | Rv0432 | Cu, Zn superoxide dismutase. Thought to be involved in detoxifying the ROI produced by macrophages | MAB_4184c | 57% | Msmeg_0835 | 66% |
| SmpB | Rv3100c | Protein involved in maintaining the bacterial translation machinery in an operational state in hostile environments | MAB_3473c | 75% | Msmeg_2091 | 76% |
| VirS | Rv3082c | Virulence regulatory factor | MAB_3997c | 35% | No | NA |
| WhiB3 | Rv3416 | Transcriptional regulator. Interacts with SigA, playing a role in virulence | MAB_3726 | 72% | Msmeg_1597 | 80% |

1. For proteins belonging to the PE/PPE and ESX families, see the text; homologs of *M. tuberculosis* LeuD, TrpD, ProC and PurC (amino acid and purine biosynthesis) present in both *M. abscessus* and *M. smegmatis* are not shown.
2. A protein homologous to IipB (Rv1478) is also present in both *M. abscessus* (MAB_2727c) and *M. smegmatis* (Msmeg_3156).
3. A protein homologous to IrtB (Rv1349) is also present in both *M. abscessus* (MAB_2261c) and *M. smegmatis* (Msmeg_6553).
4. There are three other proteins homologous to Rv3763 in *M. abscessus*: MAB_ 2379 (51% identity), MAB_3261c (35%) and MAB_4074c (34%).
5. Presence of homologs most similar to non mycobacterial proteins (see Fig. 4).
6. A cluster similar to the *narGHIJ* cluster encoding the *M. tuberculosis* nitrate reductase is present in *M. smegmatis*, but not *M. abscessus*.
7. There are three other phospholipases C encoded in H37Rv: PlcA (Rv2351c), PlcB (Rv2350c) and PlcC (Rv2349c) (see also Fig. 4).
8. Homologs of RpfB (Rv1009) and RpfC (Rv1884c) are also present in both *M. abscessus* (MAB_1130 and MAB_4080c, respectively) and *M. smegmatis* (Msmeg_5439 and Msmeg_4640, respectively).
9. Other *M. tuberculosis* sigma factors involved in virulence (SigA, SigD, SigE, SigH) have homologs in both *M. abscessus* and *M. smegmatis*.

Abbreviations: id., identity; NA, not applicable; ROI, reactive oxygen intermediates, RNI, reactive nitrogen intermediates; Mabs, *M. abscessus;* Msmeg, *M. smegmatis;* Mtb, *M. tuberculosis.*
